# Motor Synchronization in Patients With Schizophrenia: Preserved Time Representation With Abnormalities in Predictive Timing

**DOI:** 10.3389/fnhum.2018.00193

**Published:** 2018-05-16

**Authors:** Hélène Wilquin, Yvonne Delevoye-Turrell, Mariama Dione, Anne Giersch

**Affiliations:** ^1^Aix Marseille Univ, Laboratory of Clinical Psychology, Psychopathology and Psychoanalysis, Aix-en-Provence, France; ^2^SCALab, UMR 9193 – National Center for Scientific Research, University of Lille, Villeneuve d’Ascq, France; ^3^Department of Physiology, Institute of Neuroscience and Physiology, Sahlgrenska Academy, University of Gothenburg, Gothenburg, Sweden; ^4^INSERM U1114, Department of Psychiatry, Federation of Translational Medicine of Strasbourg, Strasbourg University Hospital, Strasbourg, France

**Keywords:** schizophrenia, predictive timing, sensorimotor synchronization, timing and time perception, tapping

## Abstract

**Objective:** Basic temporal dysfunctions have been described in patients with schizophrenia, which may impact their ability to connect and synchronize with the outer world. The present study was conducted with the aim to distinguish between interval timing and synchronization difficulties and more generally the spatial-temporal organization disturbances for voluntary actions. A new sensorimotor synchronization task was developed to test these abilities.

**Method:** Twenty-four chronic schizophrenia patients matched with 27 controls performed a spatial-tapping task in which finger taps were to be produced in synchrony with a regular metronome to six visual targets presented around a virtual circle on a tactile screen. Isochronous (time intervals of 500 ms) and non-isochronous auditory sequences (alternated time intervals of 300/600 ms) were presented. The capacity to produce time intervals accurately versus the ability to synchronize own actions (tap) with external events (tone) were measured.

**Results:** Patients with schizophrenia were able to produce the tapping patterns of both isochronous and non-isochronous auditory sequences as accurately as controls producing inter-response intervals close to the expected interval of 500 and 900 ms, respectively. However, the synchronization performances revealed significantly more positive asynchrony means (but similar variances) in the patient group than in the control group for both types of auditory sequences.

**Conclusion:** The patterns of results suggest that patients with schizophrenia are able to perceive and produce both simple and complex sequences of time intervals but are impaired in the ability to synchronize their actions with external events. These findings suggest a specific deficit in predictive timing, which may be at the core of early symptoms previously described in schizophrenia.

## Introduction

Many approaches, in phenomenology, neurobiology, and experimental psychology, have led to the idea that time disorders may play a role in the pathophysiology of schizophrenia. Timing disorders may affect the patients’ ability to connect to and synchronize with the outer world, possibly explaining why patients feel disconnected from reality. However, the precise mechanisms at play in this difficulty may be diverse. In the present work, a sensorimotor synchronization task (SMS) was used to investigate the importance of time interval production and synchronization abilities in patients with schizophrenia. The findings will be discussed in the light of possible clinical and practical implications of predictive-timing abnormalities in schizophrenia.

Phenomenologists have long reported that the thought flow is fragmented in time in patients with schizophrenia (Lewis, 1932; Minkowski, 1933/2005; Kimura, 1994; Fuchs, 2007, 2013; Vogeley and Kupke, 2007; Stanghellini, 2009; Mishara, 2010). Andreasen (1999) proposed the hypothesis of cognitive dysmetria (see also Friston, 2005), which is based on an analogy between the loosening of associations, the observation of cerebellum-related disorders (Feinberg and Guazzelli, 1999; Bernard and Mittal, 2014; reviews in Picard et al., 2008; Bachmann et al., 2014; Giersch et al., 2016), and the newly found role of the cerebellum in the sequencing of cognitive actions (Leiner et al., 1993; Ito, 2008; Koziol et al., 2014). Timing abilities are necessarily required to plan correctly and sequence actions through time, although the model of cognitive dysmetria does not specify the type of timing impairments involved. Recently, studies in experimental psychology have reported a difficulty in schizophrenia to discriminate between simultaneous and asynchronous information in visual perception, and to predict and expect information at the milliseconds (ms) and the second levels (Giersch et al., 2009; Lalanne et al., 2012a,b; Martin et al., 2017). For example, Martin et al. (2017) used the variable foreperiod paradigm in which a visual target is displayed at variable delays after an initial fixation point. The probability that the target is displayed increases as time passes, and subjects benefit from time passage to increase expectation and be better prepared to process the target when it is displayed. This effect typically resulted in better performance in controls for longer foreperiods. However, patients showing self-disorders (i.e., lacking the feeling of being present here and now), did not benefit from the passage of time. Furthermore, all patients were impaired when expectancy was globally weakened by the addition of catch trials, i.e., trials in which targets were absent. These findings suggest that patients have a fragility in the ability to predict the moment of appearance of a visual target, consistent with prior studies suggesting predictive timing impairments at the milliseconds level (Lalanne et al., 2012a,b).

Such a fragility may also impact the patients’ ability to interact with their environment. Predictive timing is required to decode rhythmic information and track time regularities contained within the environment, and to act in synchrony with the environment. Interacting with the environment requires indeed that motor planning takes the dynamics of perceptual information into account, like, e.g., rhythmic patterns. A fragility in the process of sensory predictions may affect this ability and impact directly the accuracy and the stability of sequential motor planning. However, other timing difficulties, such as encoding time durations, may also account for the patients’ difficulty to connect to the real world. Such difficulties have been evidenced in several studies based on duration estimation, production, reproduction and discrimination tasks (Rabin, 1957; Orme, 1966; Johnson and Petzel, 1971; Tracy et al., 1998; Volz et al., 2001; Davalos et al., 2003, 2005; Elvevag et al., 2004; Bonnot et al., 2011; Bolbecker et al., 2014). Recent meta-analyses of time perception and temporal processing in schizophrenia conclude about a heightened variance in the patients’ performances rather than a clear over- or under-estimation of interval durations (Ciullo et al., 2016; Thoenes and Oberfeld, 2017). Disturbances in encoding time intervals in patients with schizophrenia may be related to impaired executive functions in patients, rather than timing deficits *per se* (Elvevag et al., 2004; Roy et al., 2012). Nevertheless, even if timing difficulties in schizophrenia may have several origins, these temporal dysfunctions might affect the ability of the patients to benefit from regularities in the environment and thus, interact with it in a suitable way.

In the present study, the ability to connect and interact with external regularities was tested by exploring the subjects’ abilities to coordinate sequences of motor actions (finger taps) with external sequences of tones using a SMS. During SMS, the finger tapping actions must be coordinated temporally with the predictable external event, i.e., the tones, which requires both time perception and coordination abilities. Any of these abilities may be affected in patients with schizophrenia. As a matter of fact, a number of studies have evaluated SMS tasks in patients with schizophrenia (Greenwood et al., 2007; Carroll et al., 2009; Wilquin et al., 2010; Da Silva et al., 2012; Giersch et al., 2013), overall suggesting lower accuracy and greater variability in patients with schizophrenia than in controls. However, motor timing disturbances observed through SMS tasks may be of different nature, which have not yet been accurately determined.

When a tapping action must be synchronized rhythmically with an auditory metronome, it entails the production of regular time intervals, but also the anticipation of when the action must be initiated in order for it to be synchronized with the external sound. These are distinct aspects of SMS, inasmuch the production of regular intervals can coexist with asynchronies, e.g., during offbeat tapping.

The perception of ‘inter-stimuli intervals’ (ISIs) refers to the time interval between the onset of one event (tone) and the onset of its successor. The extraction of these auditory intervals is achieved by means of an automatic, implicit timing process associated to attention dynamics (Barnes and Jones, 2000; Jones et al., 2002). It does not require explicit estimation of duration. In the case of isochronous intervals, Ehrlich (1958) proposed that the SMS task refers to a specific mechanism: a self-maintained repetitive activity of initiating and executing voluntary series of movements on the basis of the extracted cadence of the series of tones. This view has been recently corroborated by findings suggesting that an isochronous sequence of tone onsets induces an underlying attentional periodicity that cyclically targets a focus of attention to expected temporal locations (Jones et al., 2002). The phenomena may be considered as the activity of a persisting and periodic process that is synchronized with an external event, which can tacitly continue despite the introduction of potential interpolated rhythms. Owing to the automatic nature of the perception of auditory sequences composed of isochronous intervals and consistent with previous findings suggesting preserved time perception abilities in schizophrenia (Carroll et al., 2009; Wilquin et al., 2010), it was hypothesized that the patients in the present study would be able to perform correctly the production of successive target intervals of time (Delevoye-Turrell et al., 2012).

However, in SMS tapping tasks, the structure of sound sequences can be more complex. In such a case, regularities in auditory arrays do not arise from successive isochronous time intervals but from, e.g., alternations between two different time intervals (300 ms and 600 ms intervals). This more complex pattern, even if metric, generates temporal expectancies guided by the explicit auditory cues detected within the auditory sequences. As proposed by the dynamic attending theory, this metrical structure with its relative “strong” and “weak” beats, modulates attentional resources over time and in turn, affects the functioning of both perceptual and motor preparatory systems (Bolger et al., 2014). Empirical findings have highlighted the complexity of alternations between two different time intervals (Fraisse, 1966; Grahn and Brett, 2007). For instance, Grahn and Brett (2007) showed that regular beats were reproduced more accurately than metric complex rhythms. In the present study, the use of complex patterns of intervals was intended to heighten the probability of evidencing impairments in rhythmic pattern extraction in the patients with schizophrenia and thus, provide the means to specify those timing mechanisms that may be at the origin of sensorimotor synchronization disturbances. The durations of the inter-response intervals (IRIs) were analyzed in addition to movement fluency and asynchrony indicators in order to assess grouping mechanisms as well as temporal anticipation.

As a matter of fact, complex auditory patterns involve perceptual organization performances that refer to the abilities to group perceptual information into coherent patterns (Silverstein and Keane, 2011). In particular, features coded close in time are likely to be bounded together. Several studies indicate dynamic grouping impairment in schizophrenia, especially in the visual domain (Uhlhaas and Silverstein, 2005; van Assche and Giersch, 2011), but also in audition (Silverstein et al., 1996). At the same time, patients are also known to have deficits in attention flexibility (Braff, 1993). These difficulties may account for accuracy loss when patients have to produce sequences of alternating time intervals (Wilquin et al., 2010; Giersch et al., 2013). In the present study, we expected such accuracy loss to be revealed by difficulties in the case of rhythmic alternations as compared to isochronous intervals.

Crucially, a performance measure was added in order to evaluate synchronization ability, i.e., the tightness of fit of sound and tap. This important aspect of functional processes underlying SMS is often forgotten in experimental assessments of studies using finger tapping tasks in patient studies. Indeed, the level of the patients’ synchronization skills is usually obtained by measuring the accuracy and the variability in the production of time intervals, consisting in the calculation of the time interval between consecutive finger taps (IRIs). However, as emphasized above, the tempo and rhythmic structure can be accurate even if the tap occurs systematically half way between two consecutive beeps. Thus, to assess the degree of synchronization capacities, it is necessary to compute asynchronies (also called synchronization errors), which are defined as the time interval between the start of each tap and the start of the corresponding tone contained within the external rhythm. Signed asynchronies, i.e., negative and positive asynchronies correspond to taps occurring before and after the beeps, respectively. They are the indicators of the ability to produce taps in synchrony with an imposed metronome. Moreover, they indicate whether the taps are anticipated or not, i.e., whether they have been planned in advance of the tone occurrence. Thus, these data provide key information about anticipatory processes in SMS, i.e., predictive timing abilities. To the best of our knowledge, no SMS studies have measured these asynchronies errors in patients with schizophrenia.

Given the recent results suggesting difficulties in time prediction, it was hypothesized that patients with schizophrenia would have difficulties in planning their actions (tap) in anticipation to the predicted moment of sound occurrence. The typical tapping task was modified slightly in order to make it more sensitive to possible anticipation impairments by including a spatial aspect to the SMS task. Indeed, our previous studies suggested that patients were more impaired at planning motor sequences than performing simple one-element motor actions (Delevoye-Turrell et al., 2007, 2012). In sum, the present study was designed to provide the means to distinguish between the role of distinct temporal difficulties when planning through space a series of voluntary movements in synchrony with an auditory rhythmic pattern. A difficulty in estimating durations should lead to imprecise and variable time intervals between successive taps. In contrast, a difficulty at anticipating the moment of occurrence of the external auditory event should mainly lead to tap-tone asynchronies. The manipulation of the type of tone sequence (isochronous, or not), allowed us to assess to what extent the difficulties observed in patients with schizophrenia are function of the necessity to extract a complex pattern of time regularities within the perceptual world.

## Materials and Methods

### Participants

Two groups participated in the current study. The first group was composed of 24 chronic outpatients aged between 25 and 57, who were diagnosed with schizophrenia (Patient group). Diagnoses were based on a structured clinical interview that was given by a single psychiatrist following the DSM-IV. Symptom severity was evaluated using the Positive and Negative Syndrome Scale (PANSS; Kay et al., 1989). All patients were recruited in the local job center (ESAT), which is associated to the Psychiatric Sector of the University Hospitals of Strasbourg. The second group was composed of 27 healthy volunteers matched for age and sex (Control group).

For both groups, exclusion criteria were substance dependence, mental retardation, history of epilepsy and physical illnesses. The present study was approved by the local ethics committee (Comité Consultatif de Protection des Personnes dans la Recherche Biomédicale d’Alsace IV) and all participants provided written informed consent prior the beginning of the study, in accordance with the recommendations of the Helsinki Declaration.

For all patients, the mean duration of illness and the mean years of education were measured. Patients were stabilized on one or more antipsychotic medications with a calculated mean daily dose in chlorpromazine equivalents. Demographics and clinical data are presented in **Table 1**.

**Table 1:** Demographic and psychometric characteristics for the patient and the control groups.

Characteristics	SZ^a^		HC^b^	Statistics	
**Demographic characteristics**	***M (SD)***		***M (SD)***	***t***	***p***
Age (years)	40.6 (8.0)		39.9 (7.7)	0.32	0.074 (ns)
Education (years)	11.45 (2.3)		14.0 (2.21)	4.03	<0.001 (s)

**Personal characteristics**	***n***		***n***		
Gender (M/F)	14/10		14/13		
Handedness (R/L)	23/1		25/2	-	-

**Clinical characteristics**	***M***	***SD***			
Positive and Negative Syndrome Scale					
Positive component	16.3	5.7			
Negative component	23.0	6.3			
General component	37.8	10.0			
Total component	77.1	18.0			
Duration of illness (years)	16.1	8.4			
Processing speed index	82.3	8.9			
Treatment measures (mean daily)					
Chlorpromazine equivalents (mg)	290	250			

*SZ, patients with schizophrenia; HC, healthy controls; s, significant; ns, non-significant.*

*^*a*^*n* = 24, ^*b*^*n* = 27.*

### Apparatus and Instructions

**FIGURE 1:**
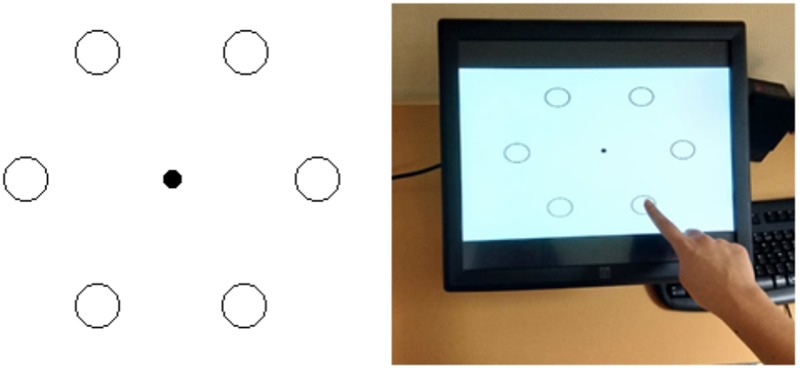
Experimental protocol. **(Left)** The left panel illustrates a top view of the tactile screen on which the picture of the targets was displayed throughout each trial. The visual targets consisted in six equidistant circles (diameter: 1.2°) arranged in the form of a hexagon. **(Right)** The right panel is a picture of a participant who was asked to point clockwise each circle, one after the other, in synchrony with a series of beeps (*N* = 30 or 34 depending on auditory sequences). These sounds were emitted by a computer and played through table-top speakers.

Participants were seated comfortably on a chair in front of a touch screen (EloTouch, 23 cm × 36 cm × 30 cm), which was placed on a narrow support at knee-height. The participants’ task was to produce a sequence of pointing movements to visual targets, with their dominant index finger, in synchrony with a series of beeps emitted by a computer. Each beep was 100 ms long and had a frequency of 333 Hz. The visual targets consisted of six equidistant circles (diameter: 1.2°) arranged in the form of a hexagon. Participants were asked to point clockwise each circle, one after the other, starting with the bottom-right circle (**Figure 1**). The trials lasted 15-s and thus, participants circled the visual pattern five or six times as a function of the type of auditory sequence to perform (*N* = 30 or 34 taps depending on isochronous or non-isochronous sequences). At the end of the trial, the visual pattern disappeared and participants could relax their hand on the side of the touch-screen. Participants were instructed to be the most accurate as possible both in space and in time throughout.

### Experimental Conditions

**FIGURE 2:**
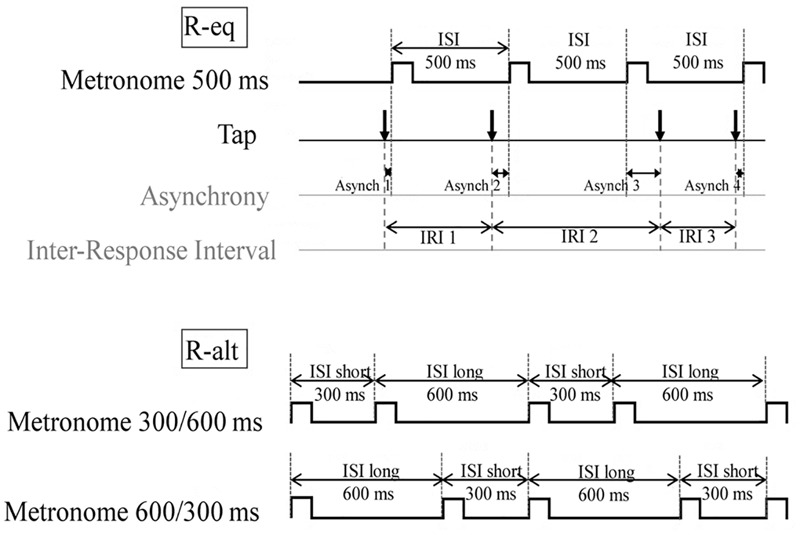
Schematic illustration of the auditory sequences and measured variables (IRI and ASYNC), which were used as indicators of the performance levels observed in the sensorimotor synchronization task. Participants were instructed to synchronize their finger-tap following two different auditory sequences. Isochronous sequences were constituted of equivalent inter-stimulus intervals (ISIs) of time (**Top**: R-eq; ISI = 500 ms). Non-isochronous sequences were composed of alternating time intervals (**Bottom**: R-alt; ISI = 300/600 ms or ISI = 600/300 ms). This figure also specifies the indicators used to characterize the participants’ timing performances. The inter-response interval (IRI) refers to the time interval between the onsets of two successive taps produced by a participant **(Top)** and reflects the participants’ ability to produce accurately a timed motor sequence. The asynchrony indicator (ASYNC) was calculated as the time interval between the first detected point of contact between the finger and the screen (i.e., the tap action) and the start of the nearest tone. It is a marker of the participants’ ability to produce a motor response in synchrony with a predictable external event. By convention, signed asynchronies are negative when the tap is ahead of the target beep (e.g., ASYNC 1), and positive when the tap lags behind (e.g., ASYNC 3). ISI, inter-stimulus interval; IRI, inter-response interval; ASYNC, asynchrony (in ms)

In the present study, the software Audacity was used to create two different auditory sequences, which are illustrated in **Figure 2**. The isochronous patterns were trials that were composed of equivalent inter-stimulus intervals (ISIs) of time, providing a regular sequence of auditory beeps (R-eq; ISI = 500 ms). The second series were composed of non-isochronous ISI patterns, i.e., trials constituted of alternating time intervals (R-alt; ISI = 300/600 ms or ISI = 600/300) affording a more complex rhythmic sequence. An overall tempo of 900 ms could be extracted from the pooled alternated short and long ISIs. The isochronous R-eq sequences were composed of 34 tones and the non-isochronous R-alt sequences were composed of 30 tones. In all conditions, the tones lasted 100 ms.

### Experimental Design

Participants were tested individually in a quiet room. They performed a familiarization phase in which they were instructed to tap to the beat of the three auditory sequences (R-eq; R-alt_300-600_; R-alt_600-300_). No feedback on performance level was provided. When participants were comfortable with the setup, instructions and equipment, they performed the test session, with each trial consisting of a *listening phase* (4.5 s), a *waiting phase without sound* (2.7 s) and a *producing phase*. The waiting phase was introduced in order to allow the participants to perceive the beat and to be ready to point to the first target at the start of the trial.

Participants performed nine trials, three trials per condition semi-randomized. Pre-analyses of the non-isochronous rhythmic sequences (R-alt_300-600_; R-alt_600-300_) revealed similar result patterns. Hence, these two trial types were collapsed for the main analysis, and will be referred to as R-alt in the following.

### Data Collection

The participants’ task was to tap clockwise around a pattern of six visual targets, tapping one circle on each metronome beat. For each participant and trial, the accuracy of motor performance was measured both in time and in space.

The spatial accuracy was considered by measuring for each trial the dispersion ellipses of the endpoints of the pointing movements performed toward each visual target. Using principal component analysis, spatial ellipses were fitted to the movement endpoint distributions (for details on the calculations, see Gordon et al., 1994). The area of each spatial ellipse (AE – calculated in mm^2^) was then computed with small values indicating better spatial accuracy (Dione and Delevoye-Turrell, 2015). To get specifications about fluency aspect of the task, the contact duration (CD) of the finger upon the tactile screen during each tap was also measured as it may be abnormal in chronic patients with schizophrenia (Delevoye-Turrell et al., 2012).

Regarding the timing aspect of the task, the following variables were considered and for better comprehension, they will be presented in two different sections: (1) producing a rhythmic pattern and (2) being synchronized to an external metronome.

#### Production Parameters

The IRI refers to the time interval between the onsets of two successive taps produced by a participant (**Figure 1**, top). This parameter, commonly used in the tapping literature (Repp, 2005), reflects the subjects’ capacity to produce accurately a timed motor sequence. Through the use of a tactile screen, the IRIs were here calculated for each trial and for each participant by detecting the first point of contact between the finger and the screen (in ms) for each pointing action.

Using the IRI measurements for each individual, the coefficient of variation (CV) was also calculated for each trial following the equation: 100^∗^IRI standard deviation/IRI mean (in %). The CV was used as an indicator of within-subject performance variability and thus, of performance stability.

#### Synchronization Parameters

The SMS is characterized by the predictability of the external beep, which arises from its regular recurrence. It is in fact this feature of predictability that allows good synchronization between own self-initiated movements and an external event. This is a clear distinction with simple reaction time tasks, for which an action is made as quickly as possible after a beep and thus, is characterized by a reaction time of 180 ms and more. Hence, the ability to anticipate the beep occurrence leads to tap-beep asynchronies close to zero or even negative asynchronies in healthy volunteers as they have been shown to have the tendency to over-estimate sensorimotor latencies (Aschersleben, 2002).

The synchronization ability of the participants in the present study was characterized by calculating an asynchrony indicator (**Figure 2**, top), which was computed for each trial and each participant. ASYNC was calculated for each tap as the time interval (in ms) between the first detected point of contact between the finger and the screen and the start of the nearest tone (Tap_Start_– Beep_Start_). This difference is referred to as the « signed asynchrony », which indicated the direction of the error of synchronization. By convention, signed asynchronies are negative when the tap precedes the target beep and positive when the tap is late. An “absolute asynchrony” was also calculated for each tap to illustrate the error amplitude of asynchronies, independently of error direction. Finally, a measure of synchronization variability was also computed. To this aim, A CV of asynchronies was calculated for each trial following the equation: 100^∗^standard deviation of Abs (ASYNC)/Abs (ASYNC) mean (in %).

### Statistical Analyses

Descriptive time series analyses were first conducted to detect possible out-liers in the tapping time series. In both groups, it took a few beeps in order for the participants to get into the rhythm set by the metronome. Hence, in the following analyses the first six taps of each trial were discarded. Analyses of variances (ANOVA) were conducted on the different dependent variables to reveal main effects of pathology and auditory sequence complexity, and interactions. These analyses were conducted with *Statistica* Software and the significance level was set to an alpha of 0.05.

## Results

Overall, participants showed no difficulties in producing series of taps in synchrony with the auditory sequences and this was true for both types of auditory sequences. The following analyses were conducted on mean CDs, with group and types of sequences as a between-group variable (since isochronous and non-isochronous sequences were run in separate blocks). The patients with schizophrenia showed significantly longer CDs (*M =* 172 ms, *SD =* 53) than controls (*M =* 140 ms, *SD =* 45); *F*(1,98) = 12.961; *p =* 0.001, $\eta_{p}^{2}$ = 0.117. All participants produced shorter CDs in non-isochronous (*M =* 137 ms, *SD =* 42) than in isochronous sequences (*M =* 174 ms, *SD =* 54); *F*(1,98) = 17.908; *p* < 0.001, $\eta_{p}^{2}$ = 0.155). No significant Group^∗^Sequence interaction effects were obtained [*F*(1,98) = 2.786; *p =* 0.098, $\eta_{p}^{2}$ = 0.028].

Spatial performances in the control group (*M =* 71 mm^2^, *SD =* 21) were more accurate than that observed in the patient group, which revealed larger areas in the spatial ellipses (*M =* 119 mm^2^, *SD =* 75), *F*(1,98) = 21.595; *p <* 0.001, $\eta_{p}^{2}$ = 0.181. Both groups were less accurate in space when producing non-isochronous sequences (*M =* 110 mm^2^, *SD =* 70) compared to isochronous sequences (*M =* 78 mm^2^, *SD =* 40); *F*(1,98) = 9.664; *p =* 0.002, $\eta_{p}^{2}$ = 0.090. The interaction Group^∗^Sequence effect was not significant [*F*(1,98) = 1.587; *p* = 0.211, $\eta_{p}^{2}$ = 0.016], suggesting a similar increase in difficulty index in both groups when synchronizing to more complex metronome sequences. These results are summarized in **Table 2**.

**Table 2:** Summary of findings obtained for the spatial accuracy and contact durations (CD in ms) in the patient and in the control groups as a function of the different rhythmic complexity conditions.

Spatial accuracy	HC	SZ	Fisher’s *F*	*p*	$\eta_{p}^{2}$
Contact duration (ms)		
Isochronous sequences (500 ms)	152 ± 47	199 ± 51			
			12.961	0.001	0.117
Non-isochronous sequences (300/600 ms)	129 ± 41	146 ± 42			
Area of ellipses (mm^2^)		
Isochronous sequences (500 ms)	62 ± 19	97 ± 49			
			21.595	<0.001	0.181
Non-isochronous sequences (300/600 ms)	81 ± 21	142 ± 90			

*SZ, patients with schizophrenia; HC, healthy controls. Analyses of variance were conducted on both contact duration and area of ellipses to reveal main effects of pathology.*

### Producing Isochronous and Non-isochronous Rhythmic Patterns

In this section, results are presented on the participants’ capacity to produce successive target intervals of time without taking into account the fact that participants were synchronized or not to the beeps.

#### Isochronous Sequences (R-eq)

The target time interval was an ISI of 500 ms. A total of 15 IRI values greater than 1000 ms (absolute value) were filtered out, leaving 93% of the observations.

**FIGURE 3:**
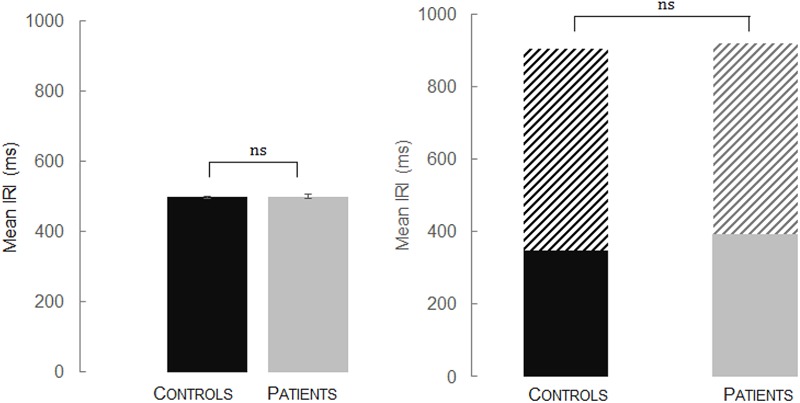
Mean inter-response intervals (IRIs in ms – with their respective standard errors) in the equivalent isochronous rhythmic condition (**Left**: R-eq) and in the alternating non-isochronous rhythmic condition (**Right**: R-alt) as a function of the group (controls; patients). For the R-alt trials, the overall tempo combines results obtained for the long intervals (600 ms – coded with hatched lines) and for the short intervals (300 ms – coded with full color).

The results from the one-way ANOVA suggested that the differences between mean IRIs in the control group (*M =* 497.9 ms, *SD =* 5.80) and in the patient group (*M =* 501.76 ms, *SD =* 11.20) were not statistically significant; *F*(1,49) = 2.431; *p =* 0.125, $\eta_{p}^{2}$ = 0.05. These results are presented in **Figure 3** (left) and indicate that all participants were able to produce accurately an isochronous auditory sequence with production of IRIs close to the 500 ms target interval.

The results from the one-way ANOVA conducted on the coefficients of variation (CV) revealed that the differences in IRI variances between the patient group (*M* = 10.13%, *SD* = 9.108) and the control group (*M* = 11.82%, *SD* = 10.00) were not statistically significant, *F*(1,49) = 0.391; *p =* 0.535, $\eta_{p}^{2}$ = 0.008. The CV values were low (around 10%) indicating an overall correct production of IRI intervals in both groups (**Table 3**).

**Table 3:** Summary of findings obtained for the coefficients of variances (CV) for the temporal variables.

	HC	SZ	Fischer’s *F*	*P*	$\eta_{p}^{2}$
CV of IRI (in %)	*M (SD)*	*M (SD)*			
Isochronous sequences	11.82 (10.00)	10.13 (9.108)	0.391	0.535	0.008
Non-isochronous sequences	27.75 (6.15)	28.28 (11.61)	0.043	0.837	0.001
CV of ASYNC (in %)	*M (SD)*	*M (SD)*			
Isochronous sequences	99.30 (24.79)	100.8 (40.25)	0.026	0.872	0.001
Non-isochronous sequences	76.38 (16.31)	76.81 (26.77)	0.005	0.945	0.001

*SZ, patients with schizophrenia; HC, healthy controls.*

#### Non-isochronous Sequences (R-alt)

The target time interval was an overall ISI of 900 ms (short and long targets pooled intervals). A total of 21 IRI values greater than 1,800 ms (absolute value) were filtered out, leaving 81% of the observations.

A one-way ANOVA was conducted on the mean IRIs. Analyses revealed an absence of Group effect, *F*(1,49) = 1.926; *p =* 0.172, $\eta_{p}^{2}$ = 0.038. The mean IRIs for the patient group (*M =* 922.38 ms, *SD =* 62.00) and the control group (*M =* 905.40 ms, *SD =* 13.78) were close to the overall target interval of 900 ms. Findings are presented in **Figure 3** (right). A one-way ANOVA conducted on the coefficients of variations (CV) did not indicate significant Group differences between the patient group (*M* = 28.28%, *SD* = 11.61) and the control group (*M* = 27.75%, *SD* = 6.15), *F*(1,49) = 0.043; *p* = 0.837, $\eta_{p}^{2}$ = 0.001 (**Table 3**). Overall, these findings indicate a preserved capacity in both controls and patients to produce non-isochronous auditory sequences.

### Maintaining Synchrony With an External Metronome

As mentioned in the data collection section, three synchronizing parameters were used to test the ability of the participants to synchronize their motor actions with an external metronome: signed asynchronies (to reveal error direction of the asynchronies: taps lagging or ahead of the metronome beep), absolute asynchronies (to indicate the amplitude of these asynchronies) and asynchrony variability (to characterize the stability of the asynchrony errors). Results for each of these measures are presented below for isochronous and non-isochronous auditory sequences, respectively. More specifically, for the non-isochronous sequences, the signed and absolute asynchronies were measured on the first tap of the overall interval of 900 ms.

#### Isochronous Sequences (R-eq)

**FIGURE 4:**
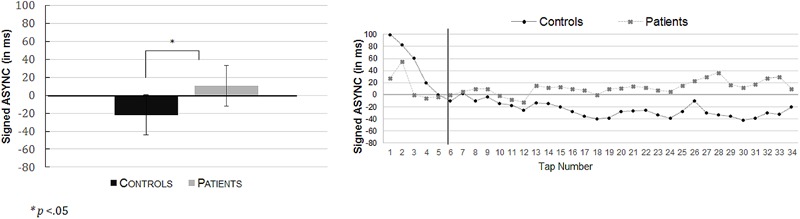
Mean signed asynchronies (ASYNC in ms) (with their respective standard errors) in the equivalent isochronous rhythmic condition (**Left**: R-eq) as a function of the group (controls; patients). On the **(Right)**, the time series of these results are presented for the total of 34 taps in controls (black dots) and in the patients (gray dots). The black vertical line illustrates the boundary from which the first six taps of each trial was discarded for the analyses. Indeed, it took a few beats for the participants to get into the pace set by the metronome.

Concerning the direction of synchronization error (signed ASYNC), a one-way ANOVA was conducted, which revealed significant differences between the control group (*M =* -21.82 ms, *SD =* 45.40) and the patient group (*M =* 10.50 ms, *SD =* 44.90). Findings indicated that controls significantly taped in advance to the beeps whereas patients were late, *F*(1,49) = 6.505; *p =* 0.014, $\eta_{p}^{2}$ = 0.117. These results are presented in **Figure 4**. The absolute ASYNC did not significantly differ between the control group (*M =* 42.97 ms, *SD =* 36.80) and the patient group (*M =* 62.21 ms, *SD =* 36.10), *F*(1,49) = 3.531; *p =* 0.066, $\eta_{p}^{2}$ = 0.067.

A one-way ANOVA conducted on the coefficients of variations (CV) of ASYNCs did not reveal significant Group differences between the patient group (*M* = 100.80%, *SD* = 40.25) and the control group (*M* = 99.30%, *SD* = 24.79), *F*(1,49) = 0.026; *p =* 0.872, $\eta_{p}^{2}$ = 0.001. The findings indicated a similar variability in synchronization performances in both controls and patients.

#### Non-isochronous Sequences (R-alt)

**FIGURE 5:**
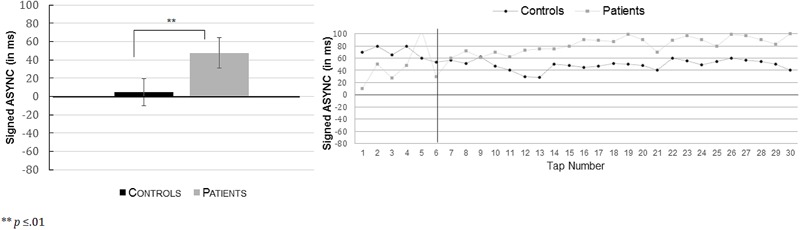
Mean signed asynchronies (ASYNC in ms) (with their respective standard errors) in the alternating non-isochronous rhythmic condition (**Left**: R-alt) as a function of the group (controls versus patients). On the **(Right)**, the time series of these results are presented for the total of 30 taps in controls (black dots) and in the patients (gray dots). The black vertical line illustrates the boundary from which the first six taps of each trial was discarded for the analyses. Indeed, it took a few beats for the participants to get into the pace set by the metronome.

The one-way ANOVA conducted on the signed ASYNC revealed significant differences between the control group (*M =* 8.23 ms, *SD =* 32.27) and the patient group (*M =* 28.27 ms, *SD* = 29.12). In both groups, asynchronies were positive but nevertheless the controls were more synchronized with the metronome than the patients, *F*(1,49) = 5.36; *p =* 0.025, $\eta_{p}^{2}$ = 0.099. These results are presented in **Figure 5**.

A one-way ANOVA conducted on the absolute ASYNC confirmed significant differences between the patient group (*M =* 76.19 ms, *SD =* 29.35) and the control group (*M =* 42.48 ms, *SD =* 15.55), indicating greater synchronization errors in the patients than in the controls, *F*(1,49) = 27.12; *p <* 0.001, $\eta_{p}^{2}$ = 0.356.

The results from the one-way ANOVA conducted on the coefficients of variations (CV) of ASYNCs revealed an absence of group differences between the patient group (*M* = 76.81 ms, *SD* = 26.77) and the control group (*M* = 76.38 ms, *SD* = 16.31), *F*(1,49) = 0.005; *p* = 0.945, $\eta_{p}^{2}$ = 0.001, indicating that the patients were no more variable in their performance than the controls.

#### Correlations

In the case of non-isochronous sequences, the Lepine disorganization score (sum of items P2, N5, G10 and G11 in the PANSS, van Assche and Giersch, 2011) correlated with the spatial area of the taps (*r* = 0.49, *N* = 24, *p* < 0.05). No other correlations were observed and in particular, there were no significant correlations between the performance scores obtained in the isochronous sequences and those coding the chlorpromazine equivalents.

## Discussion

Distortions of time have been described in schizophrenia for over a century (Lewis, 1932; Minkowski, 1933/2005). And yet, the scientific literature still gives little knowledge about the exact processes involved in the timing deficits reported in patients with schizophrenia (Ciullo et al., 2016; Thoenes and Oberfeld, 2017). Using a tapping SMS task, the results presented here were contrasted between patients with schizophrenia and controls in their abilities to produce sequences of time intervals in synchrony with a series of regular auditory tones as a function of the rhythmic complexity of the sensory information.

As in previous studies, patients revealed longer contact times than controls (Delevoye-Turrell et al., 2007, 2012), which was previously proposed as an index of the patients’ difficulty to plan voluntary motor sequences composed of (1) a downward movement of the finger, (2) a tactile surface-touching instant and (3) a lifting-up movement of the finger. The findings reported here comfort the idea that schizophrenia is characterized by a fluency difficulty in the sensorimotor integration domain of voluntary movement. The remaining of the present study was then designed to gain a better understanding of the nature of the fluency deficits related to motor and perceptual timing inabilities in schizophrenia.

The IRIs were well produced by both the controls and the patients, indicating that the cognitive abilities required for time production of both isochronous and non-isochronous intervals are preserved in schizophrenia. The relatively small amplitude of impairment in time intervals production fits with the meta-analysis of Thoenes and Oberfeld (2017) who reported that the duration-related performance was only moderately altered in patients with schizophrenia. In the present study, patients were able to take into account the tempo of the auditory metronome and produce the required inter-tap intervals, confirming a somewhat preserved perception and production of time interval durations in schizophrenia, even if the movements remained less fluent in the patients than that observed in the controls, i.e., with longer contact durations between the finger and touch-screen.

Tap-tone asynchronies were accurate in both groups. Yet only control participants were able to tap in advance of the sound and thus, showed clear anticipation as it is reported classically in the literature (for a review, see Repp, 2005). The patients, in contrast, tapped after the beep. As such, the group differences in the tap-tone asynchronies were significant indicating a clear effect in the patients. Such findings are consistent with previous results, which have been reported using a similar paradigm, albeit with more complex visual grouping features (Giersch et al., 2013). Nevertheless, the present study provided the means to gain a better understanding of the mechanisms at play in the patients’ alteration of motor prediction. Indeed, the tap-tone positive asynchronies in the patients suggest that some degree of anticipation is preserved. Indeed, a complete lack of prediction would have forced subjects to wait to perceive the sound before tapping, which would have led to positive tap-tone asynchronies greater than +100 ms (Repp and Su, 2013). This was the case in neither groups. Second, in both groups, learning curves were observed (presented in **Figures 3, 4**), which confirm the fact that participants and patients were able to detect asynchrony errors and use them to improve synchrony performances. Indeed, at the start of the trials, all participants showed a large tap-tone delay, probably because a certain number of beep intervals were needed in order to pick up the beat. Nevertheless, both groups quickly reached a plateau of tap-tone synchronies within the first six taps of a trial. It has been suggested that such adjustments do not require a conscious detection of synchrony errors (Weibel et al., 2015). Importantly, the adjustment patterns in the patients were consistent with prior results indicating that patients automatically adjust motor actions in the case of a mismatch between expected and real sensory outcomes, at least as long as the detection of this mismatch does not need conscious awareness (Fourneret et al., 2002; Delevoye-Turrell et al., 2003; Knoblich et al., 2004; Lencer et al., 2017). Another important point of these learning curves is that they suggest that the differences in tap-tone asynchronies between patients and controls appear only late, when controls start to tap in advance. Patients and controls’ performance is similar during the first beep intervals of the trials. If any, patients are rather faster than controls, suggesting that the group differences are not due to a non-specific motor slowing in the patients.

Even if negative asynchronies seem to be the golden rule for isochronous intervals, sensori-motor anticipations were less emphasized in the non-isochronous than in the isochronous rhythmic sequences in both controls and patients. Repp et al. (2011) have reported negative asynchronies even for non-isochronous intervals but these authors tested musicians only. Furthermore, Repp (2005) and Repp and Su (2013) used solely tapping paradigms for which no displacement through space was required – tapping was performed at different tempo and with different rhythmic patterns but always to a unique spatial target. A spatial aspect has been incorporated here as most voluntary motor behavior in our everyday life requires not only timing but also spatial coordination of body movements. In doing so, Giersch et al. (2013) reported that tap-tone asynchronies were always positive, comforting the results observed in the present study for which positive asynchronies were observed both in the patients and in the controls, in the non-isochronous sequences. Furthermore, the changes in the nature of the asynchronies as a function of task complexity suggest that tapping in a predictive matter is related to task attentional load. More specifically, participants may tap all the more in advance that they do the task automatically and that the synchronization parameter is adjusted on the basis of the predicted sensory consequences of action, and not on the initiation of the out-going efferent command. This explanation fits well with the results observed in musicians, who need to coordinate their actions with the sounds produced by others, and who usually show negative mean asynchronies.

Anticipatory mechanisms may be less efficient when task complexity increases. This would indicate that for the patients, even the isochronous tapping task would be effortful. This hypothesis of an increase in task difficulty (i.e., in cognitive load) in both isochronous and non-isochronous sequences in the patients is consistent with the observed increase in spatial errors, which are larger in the patients than in the controls across conditions. An increased difficulty in synchronizing taps with beats is also supported by previous work. For example, in Turgeon et al. (2012), participants were instructed to detect a shift in occurrence of a sound, presented within a series of otherwise isochronous tones. Contrary to controls, the patients had more difficulties to detect the phase shifts when they tapped in synchrony with the tones than when they simply listened to the tone series. This finding suggested a peculiar difficulty associated with SMS, with a difficulty in predictive timing abilities. This occurred in a task based on simple isochronous rhythms, which is usually believed to require only basic, automatic mechanisms. Moreover, the chosen tempo was close to the spontaneous tempo observed in adults suffering from schizophrenia (Delevoye-Turrell et al., 2012), meaning that the tempo was especially comfortable for the patients (Delevoye-Turrell et al., 2014). Hence, what is usually a very easy task for healthy controls may present a real challenge for patients with schizophrenia.

It has been proposed that the natural flow of events may have lost its continuity in schizophrenia (Martin et al., 2014; Giersch and Mishara, 2017a,b). This might account for a difficulty to benefit from the time flow when expecting an event, and to anticipate external events (Northoff and Duncan, 2016). Previous studies have suggested a link between time prediction difficulties and elementary forms of self-disorders, i.e., those enabling subjects to feel the present as being now and here. This disorder of the self usually emerges during the prodromal phase of schizophrenia, and may pre-exist full-blown symptoms (Møller and Husby, 2000; Parnas et al., 2005; Wilquin and Delevoye-Turrell, 2012). As a matter of fact, previous studies have not found correlations between the most elementary time disorders and clinical symptoms (Giersch et al., 2015). Likewise, in the present study, no correlations were found between asynchronies and clinical symptoms. A correlation with clinical disorganization was found only with spatial errors, echoing the significant correlations found between spatial organizations and clinical disorganizations (Silverstein and Keane, 2011; van Assche and Giersch, 2011). Future studies will have to investigate whether or not tap-tone asynchronies correlate with elementary forms of self-disorders in patients with schizophrenia, maybe by rendering the task more difficult through the modulation of the tempo of the sequences to produce.

## Conclusion

Our results suggest a clear dissociation between the preserved ability to produce inter-tap intervals in schizophrenia and the difficulties in timing self-initiated action to predictable external events. A dissociation between these two mechanisms in a sensori-motor synchronization task had already been reported in a study conducted in healthy adults by Palmer et al. (2014). In their study, beat-deaf patients were able to produce time intervals but were impaired in synchronizing self-produced taps with the beats of the metronome. However, contrary to patients with schizophrenia, they tapped well in advance of the sounds. The findings reported here in patients with schizophrenia thus appear to show a specific atypical pattern in synchronizing self-initiated body movements to outer world events.

## Author Contributions

AG and YD-T designed the study and edited and finalized the manuscript. AG acquired the clinical data. HW acquired the experimental data. HW, MD, and YD-T analyzed the experimental data. AG, HW, and YD-T interpreted the data. HW wrote the first draft of the manuscript. All authors reviewed, amended, and approved the manuscript.

## Conflict of Interest Statement

The authors declare that the research was conducted in the absence of any commercial or financial relationships that could be construed as a potential conflict of interest.
